# Optimising the implementation of adolescent-friendly health services and its effects on contraceptive uptake and adolescent pregnancy in rural Mozambique: an implementation research study of the S-NICE intervention

**DOI:** 10.3389/frph.2026.1828528

**Published:** 2026-06-10

**Authors:** Baltazar Chilundo, Laura Xinavane, Luísa Huo, Fátima Abacassamo, Luc Van der Veken, Benigna Matsinhe, Paula Morgado, Claudia Abreu Lopes

**Affiliations:** 1 Faculty of Medicine, Universidade Eduardo Mondlane, Maputo, Mozambique; 2 Pathfinder International, Maputo, Mozambique (Headquarters: Watertown, MA, United States); 3 National Institute of Health, Maputo, Mozambique; 4 Ministry of Health, Maputo, Mozambique; 5 International Institute for Global Health, United Nations University, Kuala Lumpur, Malaysia

**Keywords:** adolescent-friendly health services, adolescents, consolidated framework for implementation research, contraception, Mozambique, pregnancy, sexual and reproductive health

## Abstract

**Background:**

Despite national policies promoting adolescent- and youth-friendly health services, implementation gaps hinder improvements in adolescent sexual and reproductive health outcomes in Mozambique. S-NICE is a structured, participatory decision-support intervention designed to strengthen delivery through routine data use, multisectoral coordination, and local action planning. This study evaluates the implementation and effectiveness of S-NICE in rural Sofala Province.

**Methods:**

A mixed methods design integrating qualitative and quantitative approaches. The effectiveness component applied a quasi-experimental Difference-in-Difference analysis comparing eight intervention and eight comparison facilities over two six-month periods. The indicators of effectiveness were the increase in new users of modern contraceptive methods among girls aged 15–19 years and a drop in rates of adolescent pregnancy measured through attendance of first antenatal care appointment. The implementation component used a multiple case-study design guided by the Consolidated Framework for Implementation Research, drawing on 68 in-depth interviews and 21 focus group discussions (*n* = 188 participants) across health, education, community, and adolescent stakeholders to assess intervention's acceptability and appropriateness. Implementation fidelity was assessed through structured review of workshop reports.

**Results:**

Within intervention facilities, coverage of new modern contraceptive users increased from 5.45% to 10.12% (+4.67 percentage points; *p* = 0.029), while adolescent pregnancy declined from 13.76% to 11.64% (−2.12 percentage points; *p* = 0.022). However, the difference to comparison facilities did not reach statistical significance neither for contraceptive uptake (+5.21 percentage points; *p* = 0.121) nor adolescent pregnancy rates (−2.43 percentage points; *p* = 0.090). In terms of its implementation, S-NICE demonstrated moderate-to-high fidelity and strong acceptability and appropriateness across stakeholder groups. Qualitative findings indicated that participatory workshops, data-driven decision-making, and engagement of community leaders strengthened coordination and accountability, while infrastructure constraints, staff turnover, and sociocultural norms remained persistent barriers.

**Conclusions:**

S-NICE was associated with favourable trends in adolescent sexual and reproductive health outcomes and demonstrated strong implementation performance in routine health system settings. Although causal effects could not be conclusively established in a quasi-experimental study, our findings suggest that structured, data-driven, multisectoral implementation strategies may strengthen adolescent- and youth-friendly health services delivery in low-resource contexts. Longer-term and larger-scale evaluations are warranted to evaluate impact and assess sustainability.

## 1 Introduction

The Sustainable Development Goals call for universal access to sexual and reproductive health (SRH) services, including family planning, information, and education. Adolescents are a key group of interest, requiring tailored and responsive approaches that consider their specific biological, social, and developmental needs (1). To support countries in transforming these commitments into practice, the World Health Organization (WHO) has promoted Adolescent- and Youth-Friendly Health Services (AYFHS) that is a framework for improving accessibility, acceptability, equity, appropriateness, and effectiveness of care for young people (2). Following WHO's guidance, many low- and middle-income countries (LMICs), including in sub-Saharan Africa, have adopted national AYFHS policies and guidelines, over the past decade. These policies were aimed at strengthening adolescent-responsive services across facilities, schools, and communities (3–7). Despite global commitment to improve adolescent SRH, the empirical evidence indicates that, in many LMICs, effective service delivery or sustained improvements in outcomes were still not achieved (3, 4, 8). This gap between health policy investment and outcomes gains is particularly marked in sub-Saharan Africa, where adolescent SRH indicators remain poor, revealing persistent challenges in implementation of AYFHS policies and guidelines (5–7).

The clinical components of adolescent SRH care— contraceptive counselling, prevention and treatment of sexually transmitted infections, pregnancy care, and post-abortion services—are well established by scientific literature and international guidelines. Evidence suggests that under controlled or pilot conditions, AYFHS models can improve service utilisation with positive outcomes in selected SRH indicators. However, the implementation quality, coverage, and consistency of adolescent SRH services remain unequal across contexts (9, 10). When integrated into district planning, supervision, and performance management systems, their performance often declines particularly in low-resource settings and once external support is reduced or withdrawn (6). This discrepancy reflects broader implementation challenges in health systems: interventions with demonstrated efficacy frequently underperform at scale due to resource constraints, health system inefficiencies, and sociocultural dynamics (11).

Structural and contextual challenges underscore the need for implementation strategies that are responsive to local culture and institutional context. Several barriers have been consistently identified, such as inadequate provider training, limited privacy within facilities, restrictive opening hours, weak information systems, suboptimal use of routine data, and poor coordination between health, education, and community sectors (7, 12). Effective approaches must be adaptive, evidence-informed, and co-produced with local stakeholders. Rather than being imposed through stand-alone or externally driven programmes (11, 13, 14), implementation strategies must be embedded within existing governance and supervision systems to enhance implementation, sustainability, and accountability across sectors (11, 13, 14). It was in this context that global calls for learning-oriented health system strengthening approaches—emphasising governance, integration, and contextual adaptation — started to gain prominence (2).

The knowledge about effective contextual approaches is not available as scientific production related to AYFHS in sub-Saharan Africa focuses mainly on service-level inputs and adolescent health outcomes. The organisational and system-level dimensions, e.g., how district-level actors navigate, respond to, or try to resolve their challenges during routine implementation, which are decisive to explain how interventions are delivered, adapted, and sustained tend to be absent from the literature (5, 6). This reflects the reality of vertical, facility-centered, or externally supported programmes, frequently characterised by short implementation timeframes and parallel supervision, reporting and accountability mechanisms (6). This results in a limited understanding of the implementation strategies for interventions to adapt to contexts or why certain AYFHS interventions achieve greater penetration, durability, and sustainability than others.

Critically, for SRH programmes to be effective, their implementation is crucial: how these services are organised, delivered, managed, and sustained within health systems. Factors such as workforce capacity, technical infrastructure, data literacy, availability and use, governance principles, intersectoral coordination, and entrenched gender and social norms shape the fidelity, acceptability and reach of AYFHS delivery (12, 15). This helps move beyond policy formulation and supports sustainable improvements in adolescent SRH outcomes. Therefore, implementation research (IR) offers a framework for examining not only whether interventions improve SRH outcomes, but how they are implemented, which contextual determinants shape their performance, and under what conditions they can be institutionalised at scale (12, 15).

Implementation evidence is particularly important in Mozambique, a country with strong policy focus on AYFHS but with poor SRH outcomes. Adolescents constitute a substantial proportion of the national population, with approximately one quarter aged 10–19 years, and more than half under 18 (16). Recent national data indicate that adolescent pregnancy remains alarmingly high at 36%, increasing to 44% in rural areas, highlighting pronounced geographic inequities (16). In this context, sociocultural norms, poverty, and gender dynamics shape adolescent care-seeking behaviour, often exacerbating the health system's own limitations (17, 35). Early sexual debut, limited access to age-appropriate sexual information, restrictive gender norms, and weak coordination between schools and health services contribute to high levels of unintended pregnancy, sexually transmitted infections, unsafe abortion, and school dropout (18, 19).

Multiple system-level constraints continue to prevent Mozambican adolescents from accessing SRH services. These include chronic shortages of trained human resources, limited privacy in health facilities, limited-service hours, inadequate infrastructure, inconsistent recording and use of routine data, fragmented information systems, and weak intersectoral governance between the health and education sectors (19, 20). Altogether, these barriers reinforce social and gender-based vulnerabilities and undermine the reach, accessibility, and effectiveness of adolescent-responsive SRH services in Mozambique.

The Ministry of Health of Mozambique (MISAU) has issued national strategies and guidelines promoting these services, with the aim of reducing stigma, improving confidentiality, adapting service organisation, and strengthening multisectoral coordination (20). However, implementation at district and facility levels remains constrained by chronic shortages of trained human resources, limited privacy in health facilities, limited-service hours, inadequate infrastructure, inconsistent recording and use of routine data, fragmented information systems, and weak intersectoral governance between the health and education sectors (19, 20). Compounding these challenges, sociocultural norms and poverty restrain adolescents' care-seeking behaviour (7, 19).

Mozambique has made sustained investments in AYFHS since the beginning of the century. For example, the BIZ programme (in Portuguese, Programa Geração BIZ), a multisectoral initiative focused on the SRH of adolescents and young people, has been continuously rolled out nationwide without interruptions since 1999 (9, 21). Within this broader agenda, this study uses implementation research to assess the S-NICE intervention that is a component of the AYFHS programme in Mozambique, introduced by MISAU and implemented through the Improved Family Planning Initiative (IFPI; 2021–2025), a consortium led by Pathfinder International. Informed by the global Thinking Outside Separate Spaces (TOSS) approach (22), the intervention design was built on preliminary consultations with local stakeholders, which highlighted the need for approaches that respond more effectively to district-specific service bottlenecks, sociocultural dynamics, and institutional constraints.

The S-NICE implementation research study was funded by the Catalysing Policy Improvement in Africa (CPIA) programme, a consortium between the United Nations University–International Institute for Global Health (UNU-IIGH) and the International Development Research Centre (IDRC). CPIA objectives encompass enhancing SRMNCAH policy improvement, strengthening national health research and policy ecosystems, and generating generalisable lessons to inform the development of a de-colonial, nationally led, and equity-focused model of external assistance. The motivation to conduct the S-NICE implementation assessment was twofold: first, the nature of the intervention itself, which seeks to strengthen system-level decision-making and adaptation; and second, the use of an implementation-focused methodological approach to examine how the intervention operates within real-world district and facility settings.

S-NICE was designed to strengthen and contextually adapt adolescent SRH service delivery within existing district health (integrated and specific AYFHS) and education (school-health 162 corners) systems. The intervention operates through iterative cycles of participatory workshops that bring together health providers, teachers, adolescents, community leaders, and local authorities to review routine service and school data, identify bottlenecks, and jointly develop locally owned action plans. These cycles are followed by structured monitoring of implementation progress and periodic reflection meetings to assess performance and refine strategies. By embedding decision-making within routine data systems and promoting multisectoral collaboration, S-NICE aims to enhance accountability, responsiveness, and local ownership of adolescent-friendly services. The approach relies on existing data and governance structures rather than introducing parallel systems, enhancing feasibility and sustainability in resource-constrained settings.

Through iterative cycles of data review, collective prioritisation, and monitoring the services, the intervention promotes shared situational awareness, youth leadership, and adaptive learning. Unlike previous AYFHS approaches focused primarily on service delivery or demand generation, S-NICE integrates routine data review with participatory action planning within existing systems, offering a more adaptive and system-oriented implementation strategy. Community leaders and other local influencers are engaged as implementation “anchors”, supporting sociocultural responsiveness and strengthening community-level legitimacy. In doing so, S-NICE aligns with global calls for learning-oriented health system strengthening approaches that emphasise governance, integration, and contextual adaptation (2).

In this study, IR was applied to examine not only whether S-NICE improved SRH outcomes, but also how it addressed systemic bottlenecks such as data utilisation and intersectoral coordination within routine district systems. While existing studies show that AYFHS can improve service utilisation, much of this evidence comes from controlled or pilot settings. There is limited understanding of how such models perform when integrated into routine district health systems in low-resource contexts. This study addresses this gap by combining quasi-experimental evaluation with implementation research to assess both effectiveness and the processes shaping implementation in real-world settings.

This article examines S-NICE as a health system-integrated intervention implemented in resource-limited areas of Mozambique to strengthen AYFHS and improve SRH outcomes.

The objective of this study is to evaluate the effectiveness and implementation of the S-NICE intervention in strengthening AYFHS delivery, particularly by improving service access, quality, and uptake, as well as selected SRH outcomes among adolescents in rural Sofala Province, Mozambique

1. To what extent has S-NICE intervention been implemented within routine district and facility-level health and education systems, in terms of (i) fidelity or how well the procedures are followed; (ii) acceptability among stakeholders within district health and education systems; (iii) appropriateness among adolescents, health providers, teachers, community leaders, and programme stakeholders?
2. What are the contextual, organisational, and system-level barriers and facilitators influencing the implementation of S-NICE?
3. What is the effectiveness of the S-NICE intervention in improving selected adolescent SRH outcomes specifically (i) coverage of new modern contraceptive users among girls aged 15–19 years and (ii) adolescent pregnancy, measured through first antenatal care attendance among girls aged 15–19 years?

The findings aim to advance understanding of contextual challenges in strengthening adolescent SRH services and to generate lessons for national policy uptake applicable to other low-resource settings in Africa or elsewhere.

## 2 Material and methods

### 2.1 Study design

This study employed a mixed-methods design, integrating qualitative and quantitative approaches to assess both implementation quality and effectiveness of the S-NICE intervention in rural health facilities in Mozambique. The study comprised two complementary components as stated in Table 1.

**Table 1:** Design of the study with two components to assess S-NICE.

Component	Objective	Methods	Evidence produced
Implementation	To understand of how S-NICE was implemented within routine district systems. To explore contextual barriers and facilitators influencing S-NICE implementation across selected districts.	Document analysis, in-depth interviews (IDIs), and focus group discussions, (FGDs) with implementers, beneficiaries and stakeholders across health, education, and community sectors.	Feasibility, acceptance and appropriateness of S-NICE across facilities and communities. How organisational, sociocultural, and governance factors shaped implementation processes in real-world settings.
Effectiveness	To assess whether S-NICE was associated with measurable changes in adolescent sexual and reproductive health (SRH) outcomes.	Quasi-experimental design with a Difference-in-Differences (DiD) analytical approach to estimate the effect of S-NICE on selected adolescent SRH outcomes.	Changes in SRH outcomes over time between intervention and comparison facilities, thereby accounting for baseline differences and secular trends.

### 2.2 Study setting

The study was conducted in Sofala Province, central Mozambique. At the time of the study, 117 health units reported AYFHS data through the national Health Information System for Monitoring and Evaluation (SISMA). Of these, 28 health units had implemented S-NICE between July 2022 and September 2023.

For the effectiveness component, the same eight facilities with S-NICE intervention were included, alongside eight comparison facilities located in Machanga and Muanza districts (no intervention). Comparison facilities were selected based on similarity in service characteristics and catchment population size, and were situated at least 10 kilometres from intervention sites to minimise contamination.

For the implementation component, eight intervention health units from four rural districts (Búzi, Caia, Gorongosa, and Nhamatanda) were purposively selected. Selection criteria included a minimum of six months of S-NICE implementation, functional AYFHS services, and geographic diversity. This duration was considered sufficient to allow identification of implementation determinants.

### 2.3 Study period

Data collection was conducted between April and October 2024 following receipt of scientific-ethical (CIBS FM&HCM/07/2023), and administrative (307/GMS/290/024 approvals).

Quantitative data were collected retrospectively for two six-month periods: a pre-intervention period (January-June 2022) and a post-intervention period (April-September 2023).

### 2.4 Study population

The study population comprised beneficiaries, implementers, and stakeholders of the S-NICE intervention. Beneficiaries included adolescent girls and boys aged 15–19 years eligible to access AYFHS at participating facilities. Implementers and stakeholders included health providers, facility managers, teachers and school focal points, community leaders, traditional practitioners, local authorities, and S-NICE programme managers involved in or affected by the intervention.

### 2.5 Sampling and participant selection

For the effectiveness component, all available routine service data from the selected intervention and comparison health facilities during the study period were included in the analysis.

For the implementation component, purposive sampling was employed to ensure representation across key stakeholder groups and to capture diverse perspectives on S-NICE implementation. Sampling continued until thematic saturation was reached. Inclusion and exclusion criteria were defined *a priori* for each category of informant and are summarised in Table 2.

**Table 2:** Inclusion and exclusion criteria for key informants.

Informant group	Inclusion criteria	Exclusion criteria
Adolescents (15–19 years)	Aged 15–19 years; resident in facility catchment area; provided informed consent/assent	Withdrawal of consent; inability to participate due to illness or impairment; observable intoxication at interview
Health providers and facility managers (including AYFHS staff)	Assigned to adolescent SRH services at selected facility; provided informed consent	Withdrawal of consent; inability to participate due to illness; observable intoxication
Community leaders (chiefs, secretaries, *régulos*)	Resident in community ≥2 years; participated in S-NICE workshops; provided informed consent	Withdrawal of consent; inability to participate due to illness; observable intoxication
Teachers, school directors, school health focal points, ZIP coordinators	Responsible for school/adolescent health in facility catchment area; provided informed consent	Withdrawal of consent; inability to participate due to illness; observable intoxication
Traditional practitioners/matrons	Recognised practitioner residing in community ≥2 years; provided informed consent	Withdrawal of consent; inability to participate due to illness; observable intoxication

Schools were included indirectly through their linkage to the catchment areas of selected health facilities. In each intervention district, schools engaged in S-NICE workshops were purposively identified based on their active involvement in school-health activities. Schools associated with the eight selected intervention facilities were represented through teachers and school focal points participating in IDIs and FGDs.

### 2.6 Data collection and analysis

The quantitative analysis followed a DiD approach to estimate the effect of the S-NICE intervention on selected adolescent SRH outcomes (23, 24). After exploratory analyses to assess baseline comparability between intervention and comparison facilities, the primary analysis compared changes in outcome indicators over time across the two groups using t-test for independent samples. The changes within each group were analysed using t-test for paired samples. All quantitative analyses were conducted using SPSS version 28.

S-NICE workshops were district-level participatory meetings involving health providers, teachers, adolescents, community leaders, and local authorities. Participants were identified through facility and district leadership and invited based on their roles in AYFHS delivery or community engagement. Workshops followed a structured format, including routine data review, identification of bottlenecks, and joint action planning.

IDI and FGD guides were developed based on selected CFIR constructs and adapted to the local context. In line with best practices in implementation research, the CFIR framework was applied pragmatically, focusing on domains most relevant to the operational realities of rural AYFHS delivery rather than applying the framework exhaustively (15). Instruments were piloted in Portuguese and Sena, a predominant local language. Data collection was conducted by a team of trained researchers with experience in qualitative methods and adolescent SRH, under the supervision of a co-author. IDI and FGD were conducted in private settings within health facilities, schools, or community spaces, depending on participant availability and preference, to ensure confidentiality and participant comfort. Data collectors were not part of routine service delivery teams to minimise social desirability bias. Data collectors received prior training on study procedures, ethical considerations, and facilitation techniques. All interviews and discussions were audio-recorded with consent, transcribed verbatim, and translated into Portuguese where necessary.

Qualitative data were analysed using a combined deductive-inductive thematic approach. An initial coding framework was developed based on CFIR constructs, while allowing emergent themes to be identified inductively. Contextual barriers and facilitators were explored through qualitative data using a CFIR-informed coding framework, with themes mapped across key CFIR domains including intervention characteristics, inner setting, outer setting, individual characteristics, and implementation processes. Two trained researchers independently coded transcripts using Atlas.ti software. Discrepancies were resolved through discussion and consensus. Triangulation across interviews, focus groups, workshop observations, and document review enhanced credibility and trustworthiness.

Routine service data were extracted from AYFHS registers and antenatal care (ANC) registers. Variables included: (i) number of adolescent clients (15–19 years) attending AYFHS; (ii) number of new users of modern contraceptive methods (disaggregated by method type); and (iii) number of adolescent girls attending first ANC visit. These data were aggregated at facility level for pre- and post-intervention periods.

### 2.7 Study outcomes

Effectiveness outcomes were: (i) coverage of new modern contraceptive users among girls aged 15–19 years, defined as the proportion of adolescent girls attending AYFHS who initiated a modern contraceptive method during the reporting period; and (ii) adolescent pregnancy measured as the proportion of girls aged 15–19 years among all first ANC visits recorded at each facility during the specified time period.

Implementation outcomes were defined based on Proctor et al. (25). Fidelity was assessed using a structured checklist capturing adherence to planned activities, stakeholder participation, and completion of action plans. Acceptability was assessed qualitatively through stakeholder perceptions of satisfaction, perceived usefulness, and willingness to engage. Appropriateness was assessed through perceived relevance, compatibility, and fit of S-NICE within local health and community systems, derived from thematic analysis of IDIs and FGDs.

## 3 Results

### 3.1 Study participants

A total of 68 in-depth interviews (IDIs) and 21 focus group discussions (FGDs) (*n* = 188 participants) were conducted across stakeholder groups (Table 3). Adolescents represented the largest group of participants. Sociodemographic characteristics of adolescent participants are presented in Table 4.

**Table 3:** Distribution of study participants by data collection method and stakeholder group.

Stakeholder group	IDIs (n)	FGDs (n)	Total participants across IDIs and FGDs
Adolescent girls (15–19)	13	70	83
Adolescent boys (15–19)	10	58	68
Health providers (AYFHS/MCH)	10	–	10
Facility managers	6	–	6
Teachers/school focal points	7	–	7
ZIP coordinators	1	–	1
Community leaders	3	60	63
Traditional practitioners/matrons	4	–	4
APS	2	–	2
Local authorities	7	–	7
Implementers	5	–	5
**Total**	**68**	**188**	**256**

Bold values indicate total study participants.

**Table 4:** Sociodemographic characteristics of adolescent participants.

Characteristic	IDIs (*n* = 23)	FGDs (*n* = 126)
Age group		
15–17 years	52.2%	46.0%
18–19 years	47.8%	54.0%
Sex		
Female	56.5%	55.6%
Male	43.5%	44.4%
Education level		
8th–10th grade	52.2%	43.7%
11th–12th grade	47.8%	56.3%
Marital status		
Single	91.3%	80%
Married/union	8.7%	15%
Missing	–	5%

### 3.2 Effectiveness of the S-NICE intervention on adolescent SRH outcomes

The effectiveness evaluation assessed two primary outcomes among girls aged 15–19 years: (i) coverage of new modern contraceptive users among adolescent girls attending SAAJ services, and (ii) adolescent pregnancy, measured as the percentage of girls aged 15–19 years identified at first ANC attendance.

The DiD estimator quantified the additional change attributable to S-NICE as:

$$\mathrm{DiD}=(Y_{\mathrm{Intervention}}{,}_{\mathrm{Post}}-Y_{\mathrm{Intervention},\mathrm{Pre}})-(Y_{\mathrm{Comparison},\mathrm{Post}}-Y_{\mathrm{Comparison},\mathrm{Pre}})$$

where Y represents the percentage coverage of the outcome of interest. Coverage indicators were calculated as proportions relative to the relevant adolescent service population within each facility. This DiD approach isolates the estimated intervention effect by accounting for baseline differences and secular trends affecting both groups.

To complement the DiD analysis, paired t-tests were conducted to assess within-group changes from pre- to post-intervention periods. While the DiD analysis provided the primary estimate of intervention effect, within-group tests offered supplementary evidence of temporal change.

Robustness checks were performed by adjusting for available facility-level and demographic characteristics where data permitted. Statistical significance was considered at the 5% level.

#### 3.2.1 Modern contraceptive uptake

During the study period, 4,110 adolescents accessed AYFHS across intervention and comparison facilities, of whom 65.2% were female (*n* = 2,685). Among adolescent girls attending services, 74% were new users of modern contraceptive methods. The most commonly adopted method was the oral contraceptive pill (45%), followed by implants (22.7%), injectables (13%), and intrauterine devices (2%).

In intervention facilities, coverage of new female contraceptive users aged 15–19 years increased from 5.5% in the pre-intervention period to 10.1% in the post-intervention period, representing an absolute increase of 4.7 percentage points [t(6) = −3.149, *p* = 0.020]. In comparison facilities, coverage decreased 0.54 percentage points from 10.1% to 9.6%; [t(6) = −0.389, *p* = 0.710].

The DiD estimator indicated a net increase of 5.2 percentage points. Notwithstanding the positive trend, this estimate did not reach statistical significance (*p* = 0.121), suggesting insufficient evidence to confirm a significant effect attributable to S-NICE within the available sample size and post-intervention period (Table 5).

**Table 5:** Intervention effects on adolescent SRH outcomes (girls 15–19 years).

Outcome	Group	Pre	Post	Change (Post–pre)	DiD (pp)	*p*-value
New modern contraceptive users (%)	Intervention	5.45	10.12	+4.7		
	Comparison	10.13	9.59	−0.54	+5.21	0.121
Adolescent pregnancy	Intervention	13.76	11.64	−2.12		
	Comparison	21.20	21.51	+0.31	−2.43	0.09

pp, percentage points.

#### 3.2.2 Adolescent pregnancy

A total of 3,086 pregnant adolescents aged 15–19 years attended their first ANC visit during the study period in the selected facilities. In intervention facilities, adolescent pregnancy declined from 13.8% in the pre-intervention period to 11.6% in the post-intervention period, corresponding to a reduction of 2.1 percentage points [t(6) = 1,011; *p* = 0,035]. In comparison facilities, the proportion increased slightly from 21.2% to 21.5%, representing a 0.3 percentage-point increase [t(6) = 0,587; *p* = 0.576].

The DiD analysis estimated a relative reduction of 2.4 percentage points in adolescent pregnancy in intervention facilities compared with comparison facilities. Although directionally favourable, this estimate did not reach conventional statistical significance [t(3) = 6,70;*p* = 0.090] (Table 5).

Overall, statistically significant improvements were observed in intervention facilities, while no significant changes were observed in comparison facilities. Within-group paired t-tests indicated statistically significant improvements in contraceptive uptake and reductions in adolescent pregnancy in intervention facilities, whereas no significant changes were observed in comparison sites and respectively.

As the DiD estimates—which adjust for baseline differences and secular trends—did not reach statistical significance for either outcome, these findings suggest favourable trends associated with S-NICE, but insufficient statistical power. Therefore, we cannot conclusively attribute these changes to the S-NICE intervention within the study timeframe.

### 3.3 Implementation research of S-NICE intervention

Since the effectiveness of S-NICE was not conclusively established, it is crucial to explore its implementation challenges to derive recommendations to strengthen the intervention. As such, three implementation research outcomes were selected—fidelity, appropriateness, and acceptability. The implementation research study was guided analytically by the CFIR (15) which conceptualises implementation determinants across five domains: intervention characteristics, roles and characteristics of individuals, implementation process (activities and strategies), inner setting (health facilities, school), and outer setting (e.g., hospital system, school district).

#### 3.3.1 Fidelity of S-NICE

Fidelity of the S-NICE intervention was assessed using a structured checklist based on a systematic review of workshop reports, as described in the Methods section. The assessment focused on adherence to planned activities and quality of implementation, including: (i) frequency of workshop implementation as planned; (ii) coverage of core programme content; (iii) participation of expected stakeholder groups; (iv) availability of action plans endorsed by local leadership; and (v) alignment of action plans with the objectives of the intervention.

Of the 16 workshop reports expected during the study period, 12 were available for review. Among these, 83% of workshops (10/12) were conducted according to the planned schedule. Full coverage of all core programme components—including presentations from both the health facility and school sectors—was documented in 75% of workshops (9/12). Participation of all anticipated stakeholder groups (adolescents, community representatives, school personnel, health providers, and local leadership) was confirmed in 50% of workshops. Action plans signed by local authorities were available in 67% of cases (8/12). Importantly, all reviewed action plans demonstrated alignment with the objectives of the S-NICE intervention, indicating consistency between planning and programme intent. Overall, these findings suggest a moderate-to-high level of fidelity to the intervention protocol.

#### 3.3.2 Acceptability of S-NICE

Intervention's acceptability was explored using constructs encompassing perceived adaptability of the intervention, stakeholder knowledge and attitudes, implementation processes, and contextual influences within both inner and outer settings.

In terms of adaptability of the intervention, S-NICE was widely perceived as contextually responsive and suitable for rural district implementation. Participants consistently valued the multisectoral design of the workshops, which brought together adolescents, teachers, health providers, community leaders, and local authorities within a shared forum. This structure was described as promoting coordinated action and unified messaging. As one adolescent girl explained, “*when community leaders and health staff hear the same message, it is not just us trying to explain family planning to our parents*”. Similarly, a teacher noted that “*we sit in the same room with health providers and community influencers, and we all speak the same language*”. These accounts suggest that the integrated format enhanced coherence and strengthened the perceived relevance of the intervention.

Regarding stakeholder knowledge and attitudes, perceptions were predominantly positive, although uneven understanding of the intervention was observed. Some adolescents and providers demonstrated limited familiarity with S-NICE as a structured strategy, describing it simply as “*something about going to the SAAJ [AYFHS]*”. Despite this, attitudes towards the initiative were largely favourable. Many participants described S-NICE as beneficial and distinct from previous programmes due to its participatory nature. A community leader stated that “*S-NICE has changed the lives of our children; now they focus on school instead of early marriage*”, while a health provider reported “*seeing improvements in pregnancy prevention since implementation*”. Adolescents also described increased awareness of available services and improved experiences at the AYFHS, particularly in relation to confidentiality and respectful care. As one adolescent boy expressed, “*when they treat us with empathy and confidentiality, we feel free to return*”. These findings reflect strong affective and normative acceptability, alongside growing self-efficacy among adolescents and providers.

With respect to implementation processes, participants emphasised the clarity and simplicity of operational procedures. The use of existing routine data, rather than introducing new reporting tools, was perceived as facilitating implementation. A health provider remarked, “*there are no new registers or indicators—we use the same reports from the health facility, which makes it easier*”. Advance planning, timely communication, and structured feedback mechanisms were also cited as key facilitators. An implementer explained that “*we conduct regular evaluations and provide feedback from the central level, praising what works and adjusting what does not*”.

Finally, contextual influences within both inner and outer settings shaped the overall acceptability of the intervention. Within the inner setting, supportive leadership, transparent communication, and a positive organisational climate were identified as facilitators. Participants reported feeling heard and included in decision-making processes. However, limited financial incentives for teachers and community actors were perceived as demotivating. In the outer setting, infrastructural limitations—particularly insufficient privacy within SAAJ facilities—were frequently cited as barriers. A health provider noted, “*the space is small and does not offer the necessary privacy*”, while an adolescent suggested that boys and girls should be attended in separate spaces. Cultural norms and misconceptions about contraception, including fears of infertility, also persisted as challenges to service utilisation. Despite these constraints, strong collaboration between schools, health facilities, and community leaders was widely regarded as a major strength, contributing to community acceptance and improved adolescent engagement.

#### 3.3.3 Perceived appropriateness of S-NICE

Perceived appropriateness was reflected in stakeholders' assessment of the relevance, compatibility, and suitability of S-NICE in addressing adolescent SRH challenges within their districts. Across respondent groups, the initiative was widely regarded as timely and aligned with local priorities, particularly in contexts characterised by high rates of adolescent pregnancy and early marriage. A community leader stated that “*this S-NICE came at the right time, because teenage pregnancy is very serious in this district*”, highlighting the perceived fit between the intervention and pressing community needs.

In terms of relative advantage and compatibility, participants frequently compared S-NICE favourably with previous initiatives. Health providers noted that the strategy complemented existing AYFHS rather than duplicating them. As one provider explained, “*this initiative helps us do our job better, because now it is not only nurses talking about family planning; the whole community is involved*”. Teachers similarly described how school-based discussions stimulated demand for services, with students becoming more open in discussing sexuality and seeking referrals to health facilities. These accounts suggest that S-NICE was perceived not as an external imposition, but as an enhancement of existing systems.

The intervention was also viewed as compatible with community structures and leadership hierarchies. The involvement of traditional and local leaders was seen as essential in legitimising adolescent access to services and addressing sociocultural resistance. One adolescent boy described how a local leader intervened in a family dispute regarding early marriage, invoking both the S-NICE discussions and existing legislation to protect his sister's right to continue schooling. Such narratives indicate that stakeholders perceived S-NICE as contextually embedded and socially legitimate.

At the organisational level, participants reported a strong “*tension for change*”, acknowledging that existing adolescent SRH indicators were suboptimal and required improvement. A health provider observed that “*our indicators are not good, and this programme is helping us improve, even if gradually*”. The perceived simplicity of the intervention—particularly its reliance on routine data and structured action planning—further reinforced its appropriateness, as it was seen as feasible within existing resource constraints.

Finally, many respondents expressed a desire for the continuation and expansion of S-NICE, viewing it as a sustainable and scalable approach. Community leaders, adolescents, and providers recommended extending the initiative to additional districts and remote communities. As one leader stated, “*this programme should continue and expand, because it is producing positive results*”. Although sustainability was not formally assessed, these expressions of endorsement suggest strong perceived appropriateness and institutional alignment.

### 3.4 Implementation barriers and facilitators

To provide an integrated view of implementation performance across study sites, qualitative findings were synthesised according to key implementation domains, highlighting factors that facilitated or constrained delivery of the S-NICE intervention. These determinants operated at multiple levels, including intervention characteristics, stakeholder perceptions, implementation processes, organisational context, and broader community influences. Table 6 summarises the principal facilitators and barriers identified across these domains, drawing on evidence from interviews, focus group discussions, and document review.

**Table 6:** Summary of facilitator and barriers for implementation of S-NICE.

Implementation domain	Facilitators	Barriers
Intervention Characteristics (Adaptability & Relative Advantage)	Multisectoral workshops bringing together adolescents, schools, health providers, and community leaders Use of routine health facility data rather than new reporting tools Alignment with existing AYFHS services Perceived improvement in pregnancy prevention and school retention	Infrequent workshops Limited adolescent participation (particularly male adolescents)
Roles and Characteristics of Individuals (Stakeholder Knowledge & Attitudes)	Positive perceptions of impact on adolescent wellbeing Increased awareness of AYFHS services among adolescents Improved perceptions of confidentiality and respectful care Strong sense of ownership among community leaders and providers	Limited understanding of S-NICE as a structured strategy among some adolescents and providers Persistent myths and misconceptions about contraception
Implementation Process	Clear operational guidance and structured planning Advance communication and logistical organisation Regular feedback and review meetings Community leaders acting as implementation “anchors”	High turnover of trained AYFHS providers Disruptions requiring repeated reorientation of staff
Inner Setting (Organisational Context)	Supportive leadership and positive working climate Transparent communication between sectors Availability of workshop resources Alignment with adolescent protection policies (e.g., Law 19/2019)	Lack of financial or material incentives for teachers and community actors Limited staffing (e.g., single nurse covering multiple services) Transport shortages for outreach to remote areas
Outer Setting (Community & Structural Context)	Strong collaboration between schools, health facilities, and community leadership Social legitimacy through involvement of local leaders	Inadequate AYFHS infrastructure (limited privacy, shared consultation rooms) Cultural norms supporting early marriage Parental resistance to contraceptive use

## 4 Discussion

This mixed-methods implementation research study examined the effectiveness as well as fidelity, acceptability, and appropriateness of the S-NICE intervention in strengthening AYFHS in rural Sofala Province, Mozambique. By integrating quantitative outcome assessment with qualitative analysis informed by the CFIR, the study provides insight into both observed service-level changes and the multi-level processes through which these changes occurred within routine health system settings.

The findings indicate that S-NICE achieved moderate-to-high implementation fidelity and was widely accepted across stakeholder groups, including adolescents, health providers, teachers, and community leaders. Within intervention facilities, statistically significant improvements were observed in the coverage of new modern contraceptive users among adolescent girls aged 15–19 years, alongside significant reductions in adolescent pregnancy rates measured through first ANC attendance. However, DiD estimates—which adjust for baseline differences and secular trends—did not reach statistical significance for either outcome. These findings therefore suggest favourable trends associated with S-NICE while warranting cautious interpretation regarding causal attribution.

These results align with prior evidence indicating that AYFHS models can improve service utilisation and selected SRH outcomes when effectively implemented, particularly in low- and middle-income countries (9, 10, 26, 27).

### 4.1 Interpretation through an IR Lens

Application of the CFIR framework enabled a structured examination of the multi-level determinants shaping S-NICE implementation (15). At the level of intervention characteristics, simplicity and adaptability emerged as key facilitators. This finding is consistent with IR demonstrating that interventions requiring minimal disruption to existing workflows are more likely to be adopted and sustained in resource-constrained health systems (10, 12).

At the level of individual characteristics, health providers' motivation and confidence were important facilitators of implementation, while variability in knowledge and skills—exacerbated by staff turnover—highlighted the need for continuous training and supportive supervision. Similar findings have been reported in Ethiopia and Nigeria, where provider capacity and continuity were shown to influence adherence to adolescent SRH guidelines (28, 29).

Regarding the implementation process, structured use of routine service data during workshops promoted shared problem-solving and local ownership, reinforcing calls for data-driven decision-making as a core component of health system strengthening (2). By embedding decision-support processes within routine monitoring systems, S-NICE aligns with global recommendations for adaptive and learning-oriented implementation approaches (30, 31).

Within the inner setting, managerial support and a positive organisational culture were critical enablers. Facilities where leadership actively prioritised adolescent health and supported collaboration across health, education, and community sectors demonstrated stronger implementation fidelity. Conversely, infrastructure constraints—particularly limited space to ensure privacy, shortages of trained staff, and inflexible service hours—mirrored barriers widely reported in adolescent SRH programmes across sub-Saharan Africa (7, 27).

The outer setting exerted both enabling and constraining influences. Awareness and enforcement of the Adolescent Protection Law (Law No. 19/2019) among community leaders contributed to a supportive normative and policy environment, consistent with evidence that legal and policy frameworks can legitimise adolescent SRH services and facilitate community acceptance (32, 33). At the same time, persistent sociocultural norms and misconceptions regarding contraception—particularly fears related to infertility—continued to shape adolescents' and parents' decision-making, reflecting challenges documented in other African settings (7, 29).

### 4.2 Linking implementation processes to quantitative outcomes

The observed improvements in contraceptive uptake and reductions in adolescent pregnancy within intervention facilities are plausibly linked to the implementation processes identified through qualitative analysis. Participatory workshops, joint action planning, and routine review of local data likely strengthened coordination between health facilities, schools, and communities—mechanisms previously associated with improved adolescent SRH outcomes (9, 10, 34).

The absence of statistically significant DiD effects should be interpreted in light of contextual and methodological considerations. Importantly, IR recognises that complex interventions often produce incremental, context-dependent effects that may not be fully captured through quasi-experimental designs alone (12). The convergence of favourable within-facility quantitative trends with strong qualitative evidence therefore strengthens confidence in the contribution of S-NICE, while underscoring the need for longer-term evaluation.

### 4.3 Comparison with existing evidence

The findings are consistent with regional and global evidence demonstrating that adolescent-friendly health services can increase contraceptive use and reduce unintended pregnancy when supported by community engagement, provider training, and enabling policies (10, 27, 32). However, the persistence of infrastructure and human resource constraints observed in this study reflects systemic challenges reported across sub-Saharan Africa, suggesting that such barriers require system-wide solutions rather than isolated programme adjustments (7).

Compared with interventions focused primarily on demand creation or provider training, S-NICE contributes a distinct implementation strategy centred on decision-support, local adaptation, and stakeholder co-production. This approach aligns with emerging evidence advocating for adaptive implementation strategies that respond dynamically to local context rather than relying on rigid service delivery models (2, 9).

### 4.4 Policy and programme implications

This study has several implications for policy and practice. First, strengthening adolescent SRH outcomes requires investments not only in service availability but also in implementation capacity, including leadership engagement, routine data use, and cross-sector collaboration. Embedding decision-support platforms within routine district systems may enhance accountability, multisectoral coordination, and local ownership.

Second, addressing structural constraints—such as inadequate infrastructure, inflexible service hours, and staff turnover—is essential to maximise the effectiveness of adolescent-friendly service models (7, 12). Strengthening retention of trained providers and ensuring consistent adolescent engagement are particularly critical.

Third, routine data use should be institutionalised as part of programme governance. Structured reflection on service indicators can support adaptive learning and context-responsive improvements in service delivery.

Scaling up S-NICE or similar implementation strategies should therefore be accompanied by stable financing, strengthened human resources, and integration of implementation support within routine health system governance. Future evaluations incorporating cost and sustainability analyses would further inform national scale-up decisions (2).

## 5 Limitations

Several limitations should be considered when interpreting the findings of this study.

First, the quantitative component relied on routine service data extracted from health facility registers. Although such data are valuable for assessing real-world programme performance, they may be affected by incomplete recording, reporting errors, or variability in data quality across facilities. These limitations may have influenced estimates of service utilisation and adolescent pregnancy rates.

Second, the quasi-experimental DiD design, while appropriate for evaluating interventions implemented under routine conditions, cannot fully eliminate the possibility of residual confounding. The relatively small number of health facilities, short follow-up period, and reliance on aggregate facility-level data likely reduced statistical power to detect net intervention effects. The pattern of statistically significant within-facility improvements combined with non-significant DiD estimates should therefore be interpreted cautiously. It is possible that observed favourable trends reflect early-stage implementation effects that require longer follow-up to demonstrate robust causal impact.

Third, although qualitative findings were analysed across stakeholder groups, the study did not include a structured comparative analysis explicitly examining differences in perspectives between key actors. Adolescents, health providers, teachers, and community leaders represent strategically positioned groups whose interests, incentives, and normative orientations may not be fully aligned. Treating stakeholder perspectives as homogeneous may obscure important divergences in priorities, power dynamics, and accountability structures. Future analyses would benefit from a dedicated comparative table summarising major areas of convergence and divergence across stakeholder groups to better capture these dynamics.

Fourth, the study was conducted in selected rural districts of Sofala Province, which may limit the generalisability of findings to other regions of Mozambique or to urban settings characterised by different health system capacities and sociocultural dynamics. In addition, the quantitative analysis focused primarily on in-facility service utilisation and pregnancy outcomes and did not capture adolescents who did not seek care at health facilities.

Fifth, the qualitative component involved discussions of sensitive topics related to SRH, which may have introduced social desirability bias despite efforts to ensure confidentiality and create a safe interview environment. Moreover, most adolescent participants were enrolled in school, potentially under-representing the experiences of out-of-school adolescents, who may face distinct structural and social barriers to accessing services.

Finally, the study did not include an economic evaluation or formal assessment of long-term sustainability. Consequently, conclusions regarding the cost-effectiveness, scalability, and durability of the S-NICE intervention remain limited. Future research incorporating longitudinal follow-up, economic analyses, and explicit sustainability assessment would strengthen the evidence base for potential national scale-up.

## 6 Final remarks and implications for practice

This mixed-methods implementation research study suggests that S-NICE is an acceptable and contextually appropriate strategy for strengthening AYFHS in rural Mozambique. Implementation was associated with improvements in contraceptive uptake and reductions in adolescent pregnancy within intervention facilities. However, DiD estimates did not reach statistical significance, indicating that findings should be interpreted cautiously.

Overall, the convergence of quantitative trends and qualitative evidence suggests that S-NICE represents a promising implementation strategy for strengthening adolescent SRH services in routine health system settings. Further research is needed to assess long-term impact, scalability, and sustainability.

## 7 Implications for future research

Further research should incorporate longer follow-up periods, larger samples of facilities, and economic analyses to assess cost-effectiveness and sustainability. Expanding evaluations to include out-of-school adolescents and diverse geographic settings will be important to better understand equity and contextual variation in programme effects. These approaches would help address current limitations related to statistical power, generalisability, and the short observation period.

## Data Availability

The dataset is not publicly available online due to ethical and confidentiality considerations but can be made available upon reasonable request to the first author via email.
